# Association of Serum Antioxidant Minerals and Type 2 Diabetes Mellitus in Chinese Urban Residents

**DOI:** 10.3390/antiox12010062

**Published:** 2022-12-28

**Authors:** Jingjing He, Fangyan Chen, Sitong Wan, Yongting Luo, Junjie Luo, Shuli He, Daizhan Zhou, Peng An, Ping Zeng

**Affiliations:** 1 Department of Nutrition and Health, China Agricultural University, Beijing 100193, China; 2 The Key Laboratory of Geriatrics, Beijing Institute of Geriatrics, Institute of Geriatric Medicine, Chinese Academy of Medical Sciences, Beijing Hospital/National Center of Gerontology of National Health Commission, Beijing 100730, China; 3 Department of Clinical Nutrition, Peking Union Medical College Hospital, Chinese Academy of Medical Sciences, Beijing 100730, China; 4 The Second Affiliated Hospital, Zhejiang University School of Medicine, Hangzhou 310016, China

**Keywords:** zinc, copper, selenium, type 2 diabetes mellitus

## Abstract

Antioxidant minerals including zinc, copper and selenium play critical roles in the maintenance of the redox balance in the body. However, their influences on type 2 diabetes mellitus (T2DM) are still inconclusive in Chinese populations. To elucidate the relationship between antioxidant minerals and T2DM, serum zinc, copper and selenium concentrations were measured in 1443 Chinese urban residents using a 1:2 matched case-control study. Conditional logistic regression models (CLR) were used to obtain the odds ratios (ORs) and 95% confidence intervals (CIs), and restricted cubic splines (RCS) were used to examine their dose–response associations. Serum zinc (OR = 0.52 [0.35, 0.77]) and copper concentrations (OR = 0.25 [0.17, 0.37]) were negatively associated with T2DM in a fully adjusted model. An L-shaped zinc-T2DM association (*P*_overall association_ = 0.003, and *P*_nonlinearity_ = 0.005) and a negative linear copper-T2DM association (*P*_overall association_ < 0.0001, and *P*_nonlinearity_ = 0.395) were observed. No association was found between serum selenium and T2DM in fully adjusted CLR or RCS models. In addition, joint associations with T2DM were identified between serum zinc and copper and between serum selenium and copper. In conclusion, our study emphasizes the importance of an adequate intake of antioxidant minerals for T2DM prevention in the Chinese population.

## 1. Introduction

Type 2 diabetes mellitus (T2DM) is a multifactorial and polygenic metabolic disorder affecting 6.28% of the world’s population [1]. The prevalence of T2DM is still rapidly increasing in adolescents and young adults [2]. The incidence of T2DM and related complications are declining in high-income countries but have been increasing in low- and middle-income countries such as China over the past two decades [3]. In China, the prevalence of T2DM was markedly increased from 2.2 to 9.7% in adults during 2002 to 2015 [4].

The transitions of dietary patterns were regarded as key factors contributing to the fast increase in T2DM in Chinese residents [4]. A suboptimal diet is also the leading contributor to T2DM-related death or disability [5]. Dietary patterns rich in vegetables, fruit, whole grains, legumes and nuts (e.g., Mediterranean or the Dietary Approach to Stop Hypertension) are recommended as preventive or treatment approaches for T2DM [6,7]. A common feature of these foods is rich in antioxidant micronutrients, such as zinc, copper and selenium. These minerals are cofactors of several important antioxidative enzymes. The maintenance of enzymatic activities of these antioxidant molecules are essential for the maintenance of normal β-cell function and insulin sensitivity [8]. For instance, copper/zinc-dependent superoxide dismutase (Cu/ZnSOD) plays a protective role in various types of tissue by eliminating free radicals [9]. Decreased Cu/ZnSOD activity was reported in the erythrocytes of persons with T2DM [10]. Selenium is involved in the function of glutathione peroxidases (GPXs), and decreased serum GPX activity was found in T2DM persons [11].

Evidence from observational and interventional studies supported the preventive role of zinc intake for reducing the risk of T2DM [12,13]. In contrast, the role of dietary selenium or copper intake in T2DM are still controversial as heterogeneities existed in findings across studies [14,15,16]. To precisely elucidate the relationship between antioxidant minerals and T2DM, the serum or plasma levels of these antioxidant minerals were investigated in recent studies [17,18,19]. However, the limited number of studies and a relatively small sample size led to inconsistent and inconclusive results based on the current findings. Moreover, limited evidence was obtained from the Chinese population, whose dietary and environmental exposures have undergone a rapid socioeconomic transformation. Therefore, to evaluate the relationship between serum antioxidant minerals and the occurrence of T2DM, we performed a case-control study by measuring the serum levels of zinc, copper and selenium in 1443 Chinese urban residents [20].

## 2. Materials and Methods

### 2.1. Studying Population

Shanghai residents in the present study were reported previously [20,21,22]. Briefly, based on a health registration system of local residents developed by the Centers for Disease Control and Prevention of Pudong and Baoshan Districts (Pudong CDC and Baoshan CDC, Shanghai, China), 2113 Shanghai residents with T2DM and 2458 comparable healthy controls were recruited from 6 communities in 2 districts of Shanghai from December 2006 to August 2007.

Eligible participants were residents aged from 40 to 79 years and lived locally ≥ 5 years. The T2DM patients (cases) were diagnosed according to the World Health Organization (WHO) criteria (FBG concentrations ≥ 7.0 mmol/L) [23]. Controls, with FBG concentrations < 6.1 mmol/L, were recruited from the same communities as the cases. The major exclusion criteria included current or history of severe diseases (e.g., severe psychological disorders, physical disabilities, cancer, stroke, coronary heart disease, Alzheimer’s disease, dementia, tuberculosis or other communicable diseases). Same inclusion and exclusion criteria were used for controls and persons with T2DM. All participants were surveyed by questionnaire, physical measurement and provided overnight fasting blood samples. Home interviews were conducted by trained physicians or public health workers, and anthropometric and biochemical indices were measured using a standardized protocol. Informed consent was obtained from each participant. The study protocol was reviewed and approved by the ethics committee of the Shanghai Institute for Biological Sciences, Chinese Academy of Sciences. This study adhered to the principles of the Declaration of Helsinki.

Of these participants, 2495 (including 531 patients with T2DM) had sufficient serum for minerals measurements. The present study used a 1:2 matched case-control design. A total of 481 patients with T2DM who had complete demographic and physical measurements data were selected as cases. Another 962 apparently healthy subjects, matched by gender, baseline age (±2 year), served as controls.

### 2.2. Blood Sample Collection and Elements Analysis

Details of the blood sample collection and measurements of serum ions were described previously [20,21,22,24]. Briefly, 100 μL of serum sample was placed into a 15 mL centrifuge tube coated with PFA, followed by the addition of 400 μL of ultrapure grade HNO_3_ (100 ppt, 65% *v*/*v*, Tama Chemicals, Kawasaki, Japan). Then, the centrifuge tube was placed in a water bath at 150 °C for 3 h until the solution became clear. Finally, the resulting solution was diluted to about 2 mL with ultrapure water and stored at 4 °C. Ions of the diluted solution were quantified by Agilent 7500cx inductively coupled plasma mass spectroscopy (ICP-MS) system (Agilent Technologies, Tokyo, Japan), with the G3148B ISIS system (Agilent Technologies, Tokyo, Japan) to reduce detection time and volume of sample.

A total of 17 ions were measured, including zinc, copper, selenium, which were the main focus of this study, and iron (Fe), which was included as an adjustment variable in the model, as well as other elements.

### 2.3. Measurement of Covariates

Demographic information, such as age, gender, was obtained through standardized questionnaires. We used a pair-matched design for age and gender to address these two important confounding factors. All anthropometric measurements were conducted using a standardized protocol. Height (in centimeters, cm), weight (in kilograms, kg), waist circumference (cm) and hip circumference (cm) were measured after participants took off their shoes, hats, coats and sweaters. Body mass index (BMI, kg/m^2^) was calculated as weight in kilograms divided by the square of height in meters. Experienced health workers using a conventional mercury sphygmomanometer measured blood pressure (BP) on the right arm of participants after they had sat down and rested for at least 5 min. For each subject, BP was measured two times, 30 s apart, and the average of the two readings was calculated for analysis. Hypertension was defined as SBP ≥ 140 mmHg or DBP ≥ 90 mmHg. Fasting blood glucose (FBG), hemoglobin A1c, and lipids (total cholesterol (TC), triglycerides (TG), high-density lipoprotein cholesterol (HDL-C), low-density lipoprotein cholesterol (LDL-C)) were measured enzymatically according to standard methods with a modular P800 model autoanalyzer (Roche, Mannheim, Germany) with reagents (Roche Diagnostics GmbH, Mannheim, Germany).

### 2.4. Statistical Analysis

We checked all continuous variables for normal distribution using Kolmogorov–Smirnov test and histogram. Data were reported as means (±standard deviations, SD) or medians (interquartile ranges, IQR) for continuous variables according to their distributions, and counts (percentages) for categorical variables. Independent samples *t*-test or Mann–Whitney U test was used to compare the continuous variables. The χ^2^ test was used for comparison of categorical variables.

We used conditional logistic regression models to determine the associations between serum zinc, copper, selenium levels and T2DM. Each mineral was first analyzed as categorized variable in tertiles, with the lowest tertile as the reference group. Tests for linear trends were conducted using the median value of each tertile as a continuous variable. In addition to the crude model, we constructed two additional models to sequentially adjust for confounders. The confounders were selected based on the following three points. First, the variable could theoretically/professionally be a confounding factor in the association between serum minerals and T2DM. Second, this variable is significantly associated with the outcome in the bivariate model. Third, adjusting for the variable improved the goodness of fit of the model. For example, many epidemiological studies [25,26] and meta-analyses [27,28] have described a link between serum iron metabolism indicators with T2DM. Combined with its significant association with T2DM in this study, and the improvement in model fitting after additional adjustment of serum iron, we chose iron as an adjustment variable. Model 1 was adjusted for BMI, TC, TG and systolic blood pressure (SBP). Model 2 was further adjusted for serum iron, zinc, copper and selenium concentrations. Zinc, copper and selenium were mutually adjusted, that is, when one element was analyzed, the other two were adjusted. Each mineral was then categorized as a binary variable by the median and also analyzed from crude model to adjusted model 2.

We also used restricted cubic splines (RCS) to visualize and examine the dose–response association between serum zinc, copper, selenium and T2DM on a continuous scale based on the multivariable-adjusted conditional logistic regression models. The 10th, 50th and 90th percentiles were kept as the knots, and the median was set as the reference. The SAS macro program %RCS_Reg for curve fitting was provided by Desquilbet and Mariotti [29].

In addition, we conducted subgroup analyses to evaluate the potential effect modification by age (<60 or ≥60 years), gender (men or women), BMI (<24 or ≥24 kg/m^2^), hypertension (yes or no), serum zinc (<135.0 or ≥135.0 μg/dL), copper (<129.9 or ≥129.9 μg/dL) and selenium (<16.1 or ≥16.1 μg/dL). For each serum mineral (zinc, copper and selenium), the median value was the dividing line to set it into a binary variable. We multiplied the stratified variable with the median of mineral to generate the interaction term. Heterogeneity was determined by assessing the *P*-value for interaction with the likelihood ratio test comparing models with and without interaction terms.

To quantify the joint association between minerals, we defined a combined variable with four groups for every 2 minerals (based on the binary categories of each mineral), where the combined high mineral 1 and high mineral 2 served as the reference category. We combined the results of the interaction analysis with the analysis of four categorical variables to interpret the joint interaction of two minerals.

In our analysis, the goodness of fit of model 2 was always better than that of model 1 (i.e., the model-fitting statistic, Akaike information criterion (AIC), of model 2 was smaller than that of model 1). In addition, because the results of model 1 and model 2 were generally consistent, we chose model 2 as the main result to display for the dose–response association analyses and subgroup analyses to make the results succinct.

All *p* values were two-sided, and *p* < 0.05 was considered statistically significant. All analyses were conducted using SAS 9.4 (SAS Institute, Cary, NC, USA) and SPSS for Windows version 20.0 (IBM Corporation, Chicago, IL, USA) software.

## 3. Results

### 3.1. Characteristics of the Participants

The present matched case-control analysis included 1443 participants. The characteristics of the participants by case-control status are summarized in Table 1. The mean (±SD) age of the participants was 60.8 (±8.3) years. The T2DM group had a significantly higher height, weight, BMI, waist circumference, SBP, FBG and TG and a significantly lower HDL-C than those of the non-T2DM group; however, the hip circumference, diastolic blood pressure and LDL-C were comparable between the two groups. In addition, the serum zinc, copper and selenium in the T2DM group were significantly lower than those in the controls, while the serum iron was significantly higher in the T2DM participants.

### 3.2. Independent Associations between Serum Minerals and T2DM

Table 2 presents the results of the independent associations between the serum zinc, copper and selenium and the occurrence of T2DM adjusted by potential confounding factors. When the minerals were categorized into tertiles, there were statistically negative linear associations between the serum zinc (Tertile 3 vs. Tertile 1, OR = 0.52 [0.35–0.77], *P*_trend_ = 0.001) and copper (ORs [95% CIs] from Tertiles 1 to 3: 1, 0.45 [0.32, 0.63], 0.25 [0.17, 0.37], *P*_trend_ < 0.0001) with T2DM after an adjustment for the pertinent covariates. The serum selenium was negatively related to T2DM in the crude model and Model 1 adjusted for BMI, TC, TG and SBP, but the significance disappeared after a further adjustment in Model 2 for other serum ions (Tertile 3 vs. Tertile 1, OR = 0.78 [0.55–1.10], *P*_trend_ = 0.252). When the minerals were analyzed as dichotomous variables, the results were consistent with those when they were analyzed as trichotomous variables.

**Figure 1:**
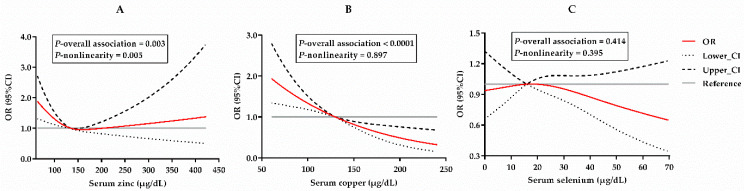
Dose–response associations between serum zinc (**A**), copper (**B**) and selenium (**C**) and T2DM. Odds ratios (ORs) and 95% confidence intervals (CIs) were derived from conditional logistic regression models. Serum zinc, copper and selenium were analyzed on a continuous scale. All the analyses were based on the adjust model 2s. Gray horizontal solid lines represent the references that were set at the median intakes, corresponding to an OR of 1.0. Red solid lines represent the multivariable-adjusted ORs, with black dotted lines representing the 95% CIs. Abbreviations: T2DM, type 2 diabetes mellitus.

Figure 1 visualizes the dose–response relationship between the serum minerals and T2DM. The RCS curves reveal a non-significant selenium-T2DM association (Figure 1C, *P*_overall association_ = 0.414) and a significantly negative linear copper-T2DM association (Figure 1B, *P*_overall association_ < 0.0001, and *P*_nonlinearity_ = 0.395). However, there was an L-shaped zinc-T2DM association (Figure 1A, *P*_overall association_ = 0.003, *P*_nonlinearity_ = 0.005), which indicated that serum zinc was inversely associated with T2DM at lower levels, while at higher levels, there was no significant association.

### 3.3. Subgroup Analysis and Joint Association

Table 3 presents the results of the subgroup analysis. We found a stronger protective effect of serum zinc levels against T2DM in older people ≥ 60 years (*P*_interaction_ = 0.027). The protective effect of serum copper was more pronounced among people ≥ 60 years (*P*_interaction_ = 0.002), with a BMI < 24 kg/m^2^ (*P*_interaction_ = 0.003) or with hypertension (*P*_interaction_ = 0.001). In addition, significant interactions existed between the minerals, namely zinc and copper (*P*_interaction_ = 0.030) and copper and selenium (*P*_interaction_ = 0.001), but not between zinc and selenium (*P*_interaction_ = 0.152). It indicated that the protective effect of serum zinc and selenium was only detected in individuals with lower serum copper levels, and copper might play a greater protective role when the level of any other mineral was low.

In Table 4, we further quantified the joint associations of the serum minerals with T2DM. The combined low serum zinc and copper was associated with the highest harmful effect (OR = 4.77 [3.35–6.79]) on T2DM compared with the combined high zinc and copper group (reference) after a full adjustment. The effects were lower for the high zinc/low copper group (OR = 2.18 [1.42–3.36]) and were nonsignificant for the high copper/low zinc group (OR = 1.08 [0.67–1.76]). The results for the joint association of copper and selenium were quite consistent with that of zinc and copper. The combined low copper/low selenium had the highest harmful effect on T2DM (OR = 3.70 [2.54–5.38]) than that of the high copper/low selenium group (OR = 0.65 [0.41–1.05]) and the low copper/high selenium group (OR = 1.86 [1.23–2.82]), compared to the high copper/high selenium group.

## 4. Discussion

In this study, using a 1:2 matched case-control design, the serum concentrations of antioxidant minerals including zinc, copper and selenium were measured in 1443 Chinese urban residents to investigate their relationships with T2DM. Serum zinc and copper concentrations were shown to be negatively associated with T2DM (Table 2 and Table 3). An L-shaped zinc-T2DM association and a negative linear copper-T2DM association were observed (Figure 1). No association was observed between the serum selenium concentration and T2DM. In addition, significant interactions were identified between the serum zinc and copper and the serum selenium and copper (Table 3). Consistently, joint associations with T2DM were also found between the serum zinc and copper and between the serum selenium and copper in Chinese urban residents (Table 4).

Elevated blood glucose and free fatty acids in T2DM patients will cause the overproduction of reactive oxygen species (ROS) by enhancing the mitochondrial oxygen consumption and activating other ROS-producing enzymes [30]. The increased ROS generation and declined capacity in endogenous antioxidant systems will impair the β-cell dysfunction and promote insulin resistance [31]. Several antioxidant molecules exist in various tissues to scavenge the ROS produced under a normal physiologic condition, including SODs, GPXs, catalases and other antioxidant micronutrients (e.g., vitamins). Metal ions zinc and copper form the center of SOD1 by catalyzing superoxide radical anions into oxygen and hydrogen peroxide in the cytosol [32]. Selenium is a part of glutathione peroxidases (GPX1-4 and GPX6), which convert peroxides to water by using reducing equivalents from glutathione in cytosol and mitochondria [14]. Therefore, adequate levels of these metal ions are required to maintain the enzymatic activities of antioxidant molecules.

Based on the data from the China National Nutrition and Health Survey 2010–2012, the dietary zinc intake of 45.8% of elderly Chinese was below the estimated average requirement (EAR) [33]. According to a survey investigating the serum zinc status in Chinese populations, the prevalence of zinc deficiency was estimated as 8.68% [34]. The major cause of a zinc deficiency is the insufficient dietary zinc intake from foods, such as oyster and beef [35]. A lower dietary zinc intake was positively associated with the occurrence of T2DM [13,36], and the dietary supplementation of zinc improved the insulin sensitivity and glycemic status [12]. However, regarding the association between the serum/plasma levels of zinc and T2DM, contradictory results were reported in Chinese populations. Some studies reported a positive association of elevated serum/plasma zinc with T2DM [37,38,39], while one study reported that an elevated serum zinc level was associated with significant protection against T2DM (OR = 0.87 [0.85–0.90]) [40]. Our results supported the protective role of serum zinc levels against T2DM (OR = 0.52 [0.35, 0.77]; Table 2). The findings from our analysis are consistent with the beneficial effects reported from observational and interventional studies of dietary zinc intake. In addition, our analysis also indicated a stronger protective effect of serum zinc on T2DM in persons ≥ 60 years (Table 3). This finding is in line with a survey result showing that zinc deficiency risk increased in elder Chinese persons [34], and the mean serum zinc concentration obtained from this nation-wide survey was comparable with that in our study (Table 1).

Findings from the China Health and Nutrition Survey indicated that dietary copper intake had no influence on T2DM [41]. Serum copper levels also appeared to have no effect on T2DM in a case-control study of 2579 adults from nine provinces of China [42]. However, our analysis indicated a protective effect of serum copper levels on T2DM (OR = 0.25 [0.17, 0.37]; Table 2) in a dose-dependent manner (Figure 1B). In addition, our analysis also identified that serum copper displayed a stronger protective effect on individuals ≥ 60 years, with a body mass index < 24 kg/m^2^ or with hypertension (Table 3). The mean concentration of serum copper in our study is comparable with those reported in Chinese populations [42,43]. Compared with previous studies, the participants in the current study are stable urban residents from one city. Moreover, a 1:2 pair-matched design for age and gender was adopted in the analysis, which further minimized the population heterogeneity. The protective effect of serum copper against T2DM is further supported by the joint analysis of serum copper and zinc (Table 4). Individuals with higher serum copper concentrations (≥129.90 μg/dL) were not sensitive to T2DM brought by lower serum zinc concentrations (<135.0 μg/dL) (Table 4). Therefore, our results suggest a sufficient serum copper concentration may benefit the glycemic status in the Chinese population.

Insufficient selenium intake from diet is still a public health concern in China. Survey data indicated that the dietary selenium intake of >50% of Chinese children and >80% of elderly Chinese was below the EAR [33,44]. Regarding the relationship between dietary selenium intake and T2DM, inconsistent findings were reported in Chinese populations [41,45,46]. Some published Chinese population studies reported that an elevated serum selenium concentration was positively associated with the occurrence of T2DM [46,47]. A comparable serum selenium concentration was found between our study and previous reports [46,47]. In contrast, in our analysis, a protective effect of serum selenium against T2DM was found in the crude linear regression model and Model 1 adjusted for BMI, TC, TG and SBP (Table 2). However, this effect on T2DM was insignificant in Model 2, further adjusted for other serum ions (Table 2). In the fully adjusted RCS analysis of the relationship, no effect was observed between the different serum selenium concentrations and T2DM (Figure 1C).

In the subgroup analysis, a significant interaction between copper and selenium was found (Table 3). Serum copper appeared to be a determinant factor that influenced the protective effect of serum selenium on T2DM. Serum selenium was only found to be protective against T2DM in individuals with low serum copper concentrations (<129.90 μg/dL), but there was no effect on individuals with high serum copper concentrations (≥129.90 μg/dL) (Table 3). This finding can be further supported by the joint analysis of the serum copper and selenium concentrations in individuals with low serum copper levels (<129.90 μg/dL) (Table 4). Our data suggest that selenium may compensate for the protective effect of serum copper on T2DM in individuals with low serum copper levels. However, as an exploratory analysis, the results of the subgroup analysis can only provide clues for further research, which needs further research to confirm.

Several limitations existed in the current study when interpreting the findings from the current analyses. First, the participants involved in this study are residents living in an urban area of southern China; thus, the conclusions drawn from these individuals could not be directly generalized to the individuals living in the rural areas or northern China due to the difference in the dietary pattern and socioeconomic status. According to the China National Nutrition and Health Survey 2010–2012, the micronutrient intake status was significantly different between big cities and rural areas and between northern and southern China [33]. Second, the participants recruited in this study are middle-aged and elderly persons; thus, the findings in this study cannot be simply generalized to young persons due to the difference in micronutrients intake and metabolic profiles. Third, the effects of serum minerals on blood glycemic parameters, such as glycosylated hemoglobin (HbA1c) levels and fasting blood insulin, were not evaluated in the current study. Further analyses between serum mineral levels and glycemic parameters will help to understand the underlying mechanisms. Fourth, serum mineral levels are not equal to the activities of related antioxidant enzymes. The measurement of related enzyme activities in T2DM patients will provide more direct evidence to answer the influences of endogenous antioxidant enzymes on glycemic status and T2DM. Fifth, the potential impact of blood glucose-, pressure- and lipid-lowering therapeutics on serum mineral levels was not analyzed in individuals with T2DM because the therapeutic information for each T2DM individual was not recorded. We cannot exclude the possibility that medications in T2DM individuals may generate an impact on plasma mineral levels, resulting in decreased zinc, copper and selenium levels. In such conditions, the proper dietary supplementation of these ions is still a feasible approach to alleviate the potential risk brought by a zinc, copper or selenium deficiency. However, the generalization of these findings to the clinical practice still requires additional validation in T2D patients receiving different therapies.

## 5. Conclusions

The current case-control study identified that serum zinc and copper were negatively associated with T2DM in urban residents of China. Selenium was shown to be negatively associated with T2DM in individuals with lower serum copper levels. Zinc and copper, and copper and selenium might have joint associations with T2DM. Our study emphasizes the importance of an adequate intake of antioxidant minerals for T2DM prevention in the Chinese population. More studies are warranted to validate our findings in populations with different socioeconomic statuses and geographical locations in China.

## Figures and Tables

**Table 1:** Characteristics of the participants by case-control status.

	Total (*n* = 1443)	Non-T2DM (*n* = 962)	T2DM (*n* = 481)	*P*
Age, years	60.8 ± 8.3	60.8 ± 8.2	60.9 ± 8.3	—
Gender				—
Men	573 (39.7)	382 (39.7)	191 (39.7)	
Women	870 (60.3)	580 (60.3)	290 (60.3)	
Height, cm	160.4 ± 7.9	159.9 ± 7.9	161.5 ± 7.7	0.0002
Weight, kg	63.3 ± 9.9	62.4 ± 9.7	65.0 ± 10.0	<0.0001
BMI, kg/m^2^	24.6 ± 3.2	24.4 ± 3.2	24.9 ± 3.3	0.005
Waist circumference, cm	83.7 ± 10.6	82.4 ± 10.8	86.4 ± 9.7	<0.0001
Hip circumference, cm	96.7 ± 10.1	96.5 ± 11.2	97.1 ± 7.6	0.239
Systolic blood pressure, mmHg	135.4 ± 19.7	132.8 ± 17.8	140.4 ± 22.2	<0.0001
Diastolic blood pressure, mmHg	81.0 ± 9.8	81.0 ± 9.5	81.1 ± 10.4	0.858
Fasting blood glucose, mmol/L	5.9 ± 2.5	4.7 ± 0.6	8.4 ± 2.9	<0.0001
HDL-C, mmol/L	1.3 ± 0.3	1.3 ± 0.3	1.2 ± 0.3	<0.0001
LDL-C, mmol/L	2.7 ± 0.7	2.7 ± 0.6	2.7 ± 0.7	0.860
Total cholesterol, mmol/L	4.8 ± 1.0	4.9 ± 0.9	4.6 ± 1.1	<0.0001
Triglycerides, mmol/L	1.4 (1.0, 2.1)	1.4 (0.9, 2.0)	1.6 (1.1, 2.3)	<0.0001
Zinc, μg/dL	140.2 ± 57.6	147.7 ± 60.6	125.2 ± 47.6	<0.0001
Copper, μg/dL	130.7 ± 41.2	139.5 ± 40.7	113.2 ± 36.2	<0.0001
Selenium, μg/dL	16.1 (11.9, 22.6)	17.6 (12.5, 23.9)	13.8 (11.3, 19.5)	<0.0001
Iron, μg/dL	548.4 (410.3, 767.7)	538.1 (404.1, 717.1)	576.7 (428.4, 910.9)	0.001

Values are presented as the mean ± standard deviations, count (%) or median (interquartile ranges). Independent samples *t*-test or Mann–Whitney U test was used to compare the continuous variables. The χ2 test was used for comparison of categorical variables. Abbreviations: BMI, body mass index; HDL-C, high-density lipoprotein cholesterol; LDL-C, low-density lipoprotein cholesterol; T2DM, type 2 diabetes mellitus.

**Table 2:** Independent associations between serum zinc, copper and selenium levels and T2DM.

				T2DM, OR (95%CI)		
Serum Minerals	*n*	Median	Cases (%)	Crude Model	Adjust Model 1	Adjust Model 2
Zinc, μg/dL						
As categorical variable (3 groups)						
Tertile 1 (<116.9)	481	96.6	244 (50.7)	1 (reference)	1 (reference)	1 (reference)
Tertile 2 (116.9 to 151.3)	481	135	130 (27.0)	0.34 (0.26, 0.46)	0.39 (0.28, 0.52)	0.54 (0.39, 0.77)
Tertile 3 (≥151.3)	481	172.8	107 (22.3)	0.26 (0.19, 0.35)	0.28 (0.20, 0.39)	0.52 (0.35, 0.77)
*P*-trend				<0.0001	<0.0001	0.001
As categorical variable (2 groups)						
Low (<135.0)	721	107.6	321 (44.5)	1 (reference)	1 (reference)	1 (reference)
High (≥135.0)	722	161.2	160 (22.2)	0.34 (0.27, 0.43)	0.36 (0.28, 0.47)	0.61 (0.44, 0.83)
*P*				<0.0001	<0.0001	0.0016
Copper, μg/dL						
As categorical variable (3 groups)						
Tertile 1 (<106.5)	481	88.5	256 (53.2)	1 (reference)	1 (reference)	1 (reference)
Tertile 2 (106.5 to <149.8)	481	129.9	143 (29.7)	0.41 (0.30, 0.56)	0.40 (0.29, 0.54)	0.45 (0.32, 0.63)
Tertile 3 (≥149.8)	481	169.8	82 (17.1)	0.19 (0.14, 0.28)	0.23 (0.16, 0.33)	0.25 (0.17, 0.37)
*P*-trend				<0.0001	<0.0001	<0.0001
As categorical variable (2 groups)						
Low (<129.9)	721	96.2	350 (48.5)	1 (reference)	1 (reference)	1 (reference)
High (≥129.9)	722	159.6	131 (18.1)	0.22 (0.17, 0.28)	0.25 (0.18, 0.33)	0.32 (0.24, 0.44)
*P*				<0.0001	<0.0001	<0.0001
Selenium, μg/dL						
As categorical variable (3 groups)						
Tertile 1 (<13.2)	481	10.5	213 (44.3)	1 (reference)	1 (reference)	1 (reference)
Tertile 2 (13.2 to <20.1)	481	16.1	157 (32.6)	0.61 (0.47, 0.79)	0.65 (0.49, 0.86)	0.87 (0.63, 1.20)
Tertile 3 (≥20.1)	481	28.3	111 (23.1)	0.37 (0.28, 0.49)	0.46 (0.34, 0.62)	0.78 (0.55, 1.10)
*P*-trend				<0.0001	<0.0001	0.252
As categorical variable (2 groups)						
Low (<16.1)	721	11.9	308 (42.7)	1 (reference)	1 (reference)	1 (reference)
High (≥16.1)	722	22.6	173 (24.0)	0.41 (0.32, 0.51)	0.48 (0.38, 0.62)	0.78 (0.58, 1.04)
*P*				<0.0001	<0.0001	0.089

Odds ratios (ORs) and 95% confidence intervals (CIs) were derived from conditional logistic regression models. Crude model, conditioned on matching factor (i.e., gender and age). Adjust model 1, further adjusted for BMI, total cholesterol, triglycerides and systolic blood pressure. Adjust model 2, further adjusted for serum iron, zinc, copper and selenium. Zinc, copper and selenium were mutual adjusted, that is, when one element was analyzed, the other two were adjusted. *P*-values for trend (P-trend) were calculated by modeling the median value of each tertile as a continuous variable. Abbreviations: BMI, body mass index; T2DM, type 2 diabetes mellitus.

**Table 3:** Subgroup analyses of the association of serum zinc, copper and selenium with T2DM stratified by age, gender, BMI, hypertension and serum minerals.

		Zinc as a Binary Variable		Copper as a Binary Variable		Selenium as a Binary Variable	
Subgroups	*n* (%)	OR (95% CI)	*P*-Interaction	OR (95% CI)	*P*-Interaction	OR (95% CI)	*P*-Interaction
Age, years							
<60	612 (42.4)	0.65 (0.41, 1.03)	0.027	0.49 (0.31, 0.77)	0.002	0.68 (0.45, 1.02)	0.723
≥60	831 (57.6)	0.52 (0.33, 0.81)		0.24 (0.15, 0.37)		0.97 (0.63, 1.5)	
Gender							
Men	573 (39.7)	0.75 (0.45, 1.24)	0.910	0.33 (0.2, 0.54)	0.788	0.83 (0.5, 1.38)	0.879
Women	870 (60.3)	0.54 (0.36, 0.81)		0.31 (0.21, 0.47)		0.72 (0.5, 1.04)	
BMI, kg/m^2^							
<24	642 (44.5)	0.52 (0.24, 1.13)	0.464	0.13 (0.06, 0.31)	0.003	0.96 (0.47, 1.95)	0.152
≥24	801 (55.5)	0.47 (0.28, 0.79)		0.58 (0.36, 0.93)		0.81 (0.5, 1.31)	
Hypertension							
No	749 (51.9)	0.82 (0.45, 1.48)	0.162	0.57 (0.33, 1.01)	0.001	0.61 (0.35, 1.07)	0.969
Yes	694 (48.1)	0.69 (0.38, 1.24)		0.10 (0.05, 0.23)		1.05 (0.61, 1.82)	
Copper, μg/dL							
<129.9	721 (50.0)	0.46 (0.27, 0.78)	0.030	-	-	0.44 (0.26, 0.76)	0.001
≥129.9	722 (50.0)	0.57 (0.29, 1.10)		-		1.07 (0.56, 2.07)	
Zinc, μg/dL							
<135.0	721 (50.0)	-	-	0.15 (0.07, 0.31)	0.030	0.67 (0.38, 1.18)	0.152
≥135.0	722 (50.0)	-		0.43 (0.24, 0.77)		0.82 (0.44, 1.51)	
Selenium, μg/dL							
<16.1	721 (50.0)	0.71 (0.39, 1.28)	0.152	0.16 (0.08, 0.34)	0.001	-	-
≥16.1	722 (50.0)	0.51 (0.28, 0.93)		0.56 (0.31, 0.99)		-	

Odds ratios (ORs) and 95% confidence intervals (CIs) were derived from conditional logistic regression models. Serum zinc, copper and selenium were analyzed as binary variables categorized by the medians. All the analyses were based on the adjust model 2s, adjusted for BMI, total cholesterol, triglycerides, systolic blood pressure, serum iron, zinc, copper and selenium. Zinc, copper and selenium were mutual adjusted, that is, when one element was analyzed, the other two were adjusted. *P*-values for interaction were assessed using likelihood ratio test comparing models with and without interaction terms. Abbreviations: BMI, body mass index; T2DM, type 2 diabetes mellitus.

**Table 4:** Joint associations of serum minerals (copper and zinc, and copper and selenium) with T2DM.

				T2DM, OR (95% CI)		
Combined Variables		*n*	Cases (%)	Crude Model	Adjust Model 1	Adjust Model 2
Copper, μg/dL	Zinc, μg/dL					
≥129.9	≥135.0	544	97 (17.8)	1 (reference)	1 (reference)	1 (reference)
≥129.9	<135.0	178	34 (19.1)	1.01 (0.64, 1.60)	1.14 (0.71, 1.84)	1.08 (0.67, 1.76)
<129.9	≥135.0	178	63 (35.4)	2.59 (1.74, 3.84)	2.35 (1.54, 3.58)	2.18 (1.42, 3.36)
<129.9	<135.0	543	287 (52.9)	5.72 (4.20, 7.80)	5.16 (3.69, 7.21)	4.77 (3.35, 6.79)
*P*-trend				<0.0001	<0.0001	<0.0001
Copper, μg/dL	Selenium, μg/dL					
≥129.9	≥16.1	504	95 (18.9)	1 (reference)	1 (reference)	1 (reference)
≥129.9	<16.1	218	36 (16.5)	0.80 (0.52, 1.25)	0.75 (0.48, 1.19)	0.65 (0.41, 1.05)
<129.9	≥16.1	218	78 (35.8)	2.38 (1.63, 3.47)	2.25 (1.51, 3.37)	1.86 (1.23, 2.82)
<129.9	<16.1	503	272 (54.1)	5.89 (4.26, 8.13)	4.77 (3.38, 6.75)	3.70 (2.54, 5.38)
*P*-trend				<0.0001	<0.0001	<0.0001

We defined combined variables with four groups for every 2 minerals (based on the binary categories of each mineral). Odds ratios (ORs) and 95% confidence intervals (CIs) were derived from conditional logistic regression models. Crude model, conditioned on matching factor (i.e., gender, age). Adjust model 1, further adjusted for BMI, total cholesterol, triglycerides and systolic blood pressure. Adjust model 2, further adjusted for serum iron, zinc, copper and selenium. Zinc, copper and selenium were mutual adjusted, that is, when one element was analyzed, the other two were adjusted. *P*-values for trend were calculated by modeling the median value of each tertile as a continuous variable. Abbreviations: BMI, body mass index; T2DM, type 2 diabetes mellitus.

## Data Availability

The data are contained within this article.
